# Secreted Amyloid Precursor Protein Alpha, a Neuroprotective Protein in the Brain Has Widespread Effects on the Transcriptome and Proteome of Human Inducible Pluripotent Stem Cell-Derived Glutamatergic Neurons Related to Memory Mechanisms

**DOI:** 10.3389/fnins.2022.858524

**Published:** 2022-05-26

**Authors:** Katie Peppercorn, Torsten Kleffmann, Owen Jones, Stephanie Hughes, Warren Tate

**Affiliations:** ^1^Department of Biochemistry, University of Otago, Dunedin, New Zealand; ^2^Brain Health Research Centre, University of Otago, Dunedin, New Zealand; ^3^Division of Health Sciences, Research Infrastructure Centre, University of Otago, Dunedin, New Zealand; ^4^Department of Psychology, University of Otago, Dunedin, New Zealand

**Keywords:** APP, amyloid beta (A4) precursor protein, sAPPα, human neuron, proteome, transcriptome, Alzheimer’s disease, memory

## Abstract

Secreted amyloid precursor protein alpha (sAPPα) processed from a parent human brain protein, APP, can modulate learning and memory. It has potential for development as a therapy preventing, delaying, or even reversing Alzheimer’s disease. In this study a comprehensive analysis to understand how it affects the transcriptome and proteome of the human neuron was undertaken. Human inducible pluripotent stem cell (iPSC)-derived glutamatergic neurons in culture were exposed to 1 nM sAPPα over a time course and changes in the transcriptome and proteome were identified with RNA sequencing and Sequential Window Acquisition of All THeoretical Fragment Ion Spectra-Mass Spectrometry (SWATH-MS), respectively. A large subset (∼30%) of differentially expressed transcripts and proteins were functionally involved with the molecular biology of learning and memory, consistent with reported links of sAPPα to memory enhancement, as well as neurogenic, neurotrophic, and neuroprotective phenotypes in previous studies. Differentially regulated proteins included those encoded in previously identified Alzheimer’s risk genes, APP processing related proteins, proteins involved in synaptogenesis, neurotransmitters, receptors, synaptic vesicle proteins, cytoskeletal proteins, proteins involved in protein and organelle trafficking, and proteins important for cell signalling, transcriptional splicing, and functions of the proteasome and lysosome. We have identified a complex set of genes affected by sAPPα, which may aid further investigation into the mechanism of how this neuroprotective protein affects memory formation and how it might be used as an Alzheimer’s disease therapy.

## Introduction

Learning and memory can be modulated by the brain protein, secreted amyloid precursor protein alpha (sAPPα), and so this molecule has potential for developing therapies preventing, delaying, or even reversing Alzheimer’s disease (AD) (Mockett et al., 2017). To harness this potential, the mechanism of how sAPPα exerts its function of preserving and protecting memory needs to be elucidated in detail.

Secreted amyloid precursor protein alpha (also referred to in the literature as sAβPPα or APPsα) is the extracellular soluble proteolytic cleavage product of plasma membrane bound APP. APP is a synaptic adhesion molecule (SAM) associated with the formation of synaptic connections during neurodevelopment, contributing to the integration of the neural network, influencing neuronal migration, and the functional organisation of the growth cone and dendritic spines, where it is enriched (Stahl et al., 2014; Sosa et al., 2017). APP acts as a WNT receptor, binding the ligands WNT3a and WNT5a and this binding in turn regulates APP protein levels (Liu et al., 2021).

Endogenous sAPPα is released extracellularly after cleavage from APP by the membrane bound alpha secretases, A Disintegrin And Metalloproteinases (ADAM) 9, 10, and 17 (also known as TACE) (Allinson et al., 2003; Asai et al., 2003).

Exogenous sAPPα enhances memory (Meziane et al., 1998), is neuroprotective (Turner et al., 2007), neurogenic (Caille et al., 2004; Baratchi et al., 2012), and neurotrophic (Chasseigneaux et al., 2011). Recombinant sAPPα, can be produced in cultured HEK cells (Turner et al., 2007), and this protein regulates gene expression (Ryan et al., 2013), stimulates synaptic protein synthesis (Claasen et al., 2009) and enhances Long-Term Potentiation (LTP) (Mockett et al., 2019) and spatial learning (Ishida et al., 1997; Taylor et al., 2008).

Few studies have analysed gene expression in isolated neurons after exposure to sAPPα. IGF2 expression was shown to be regulated by sAPPα in human neuroblastoma cells (SHSY5Y) (Morris, 2011), and in rat hippocampal neurons (Stein et al., 2004). A gene array experiment identified a set of temporally changing differentially expressed genes in hippocampal slices (Ryan et al., 2013). Lentiviral overexpression of sAPPα in a mouse model of AD identified upregulation of neuroprotective genes (Ryan et al., 2021). sAPPα specifically upregulates Arc synthesis (Livingstone et al., 2019), and glutamate AMPA receptor synthesis and its trafficking (Livingstone et al., 2021).

Here we describe, how exogenously produced sAPPα applied extracellularly to human neurons (i^3^Ns) in culture affects the cells’ transcriptome and proteome.

RNA sequencing, an established technique for identifying and quantifying changes in global expression (Chu and Corey, 2012) was used alongside Sequential Window Acquisition of All THeoretical Fragment Ion Spectra-Mass Spectrometry (SWATH-MS), which is a quantitative and highly sensitive method for assessing changes in the proteome under different conditions (Gillet et al., 2012; Collins et al., 2013).

RNA sequencing detected both protein coding (30%) and non-coding transcripts (70%), which were significantly (*p* ≤ 0.05) differentially expressed (minimum 1.5-fold change), indicative of a complex transcriptional and translational regulatory function of sAPPα that has not previously been described. SWATH-MS analysis of the differentially regulated proteins identified many that were related to neurological functions.

## Materials and Methods

### Synthesis of Secreted Amyloid Precursor Protein Alpha

The protocol for the expression of human sAPPα^695^ in HEK cells in culture and secretion into the media was developed originally in the laboratory of Warren Tate (Turner et al., 2007). Briefly, HEK cells, stably transformed with the gene fragment for sAPPα, were cultured in DMEM without serum. sAPPα was expressed and secreted into the media, and the collected media (500 mL) was concentrated by precipitation with 60% (w/v) ammonium sulphate, with stirring for 1 h at 4°C followed by centrifuging at 10,000 × *g* for 45 min. The protein precipitate was resuspended in 20 mM Tris–HCl, pH 7.0 and residual salt was first removed by FPLC coupled to a HiTrap^®^ desalting column. The post translational modifications added to sAPPα during expression in the HEK cells bind to heparin and so a Heparin Sepharose column can be used for affinity purification. The recovered protein fraction was bound to a HiTrap^®^ heparin column and sAPPα was eluted with an increasing NaCl gradient in the buffer. Salt was again removed by FPLC with a HiTrap^®^ desalting column. SDS-PAGE, Western blotting, and BCA assay analysis confirmed the purity, identity, and determined the concentration of the purified sAPPα respectively.

### Human Cortical Neuron (i^3^N) Cell Culture

**FIGURE 1:**
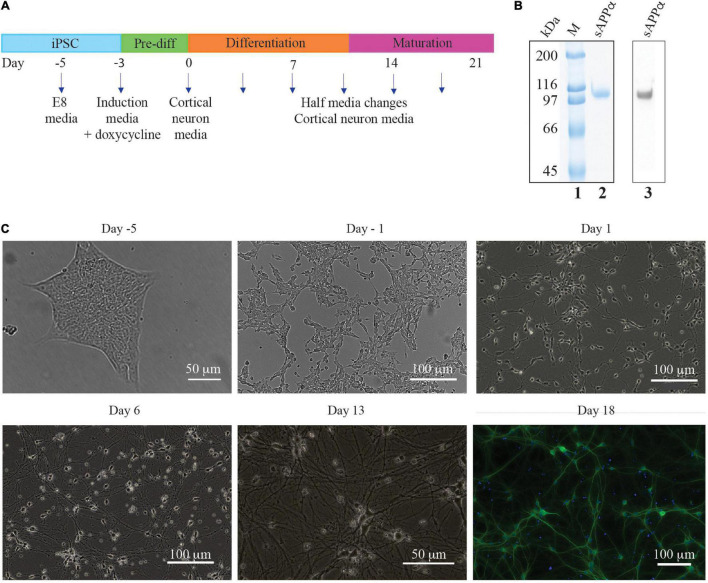
Timeline of iPSC derived neuron generation. **(A)** E8 media until differentiation was required. Three day exposure (day −3 to day 0) to induction media containing doxycycline initiated NGN2 cortical neuron transcription factor gene expression. Cells were then cultured on poly L ornithine plates in cortical neuron media. **(B)** SDS-PAGE of purified sAPPα stained with colloidal Coomassie (lane 2) and a Western blot with N-terminal APP antibody (lane 3) which confirms the identity of sAPPα. Lane 1 has broad range protein markers (Biorad), **(C)** Brightfield photographs illustrating the generation of neurons generated from an mNGN2-iPSC colony (Day −5). Initially the iPSC colony is passaged as a single cell suspension (day −3, not shown) and after 2 days exposure to doxycycline the cells have elongated and neurites beginning to develop (day −1). After 3 days exposure to doxycycline the cells are committed to the neuron fate and are plated onto poly L ornithine plates where neurites develop further and lengthen (Day 1 and 6) and make connections with other neurons (Day 13). DIV 18 i3Ns were probed with antibodies against MAP2 (dendrite).

This protocol was developed by the laboratory of Michael Ward (National Institute of Health, MD, United States) (Wang et al., 2017; Fernandopulle et al., 2018; Figure 1A). WTC11 hNGN2 inducible pluripotent stem cells (iPSC’s) were cultured in Essential 8 media (Gibco) on Matrigel™ (Corning) coated plates until required for differentiation into cortical neurons, at which time they were passaged using accutase (Gibco) to obtain a single cell suspension. Cells (3 × 10^6^) were seeded onto 100 mm Matrigel coated cell culture dishes with Induction Media (IM; DMEM), 1 × N2 supplement (Gibco), 1 × non-essential amino acids (NEAA, Gibco), 1 × GlutaMax (Gibco) supplemented with 10 μM doxycycline (dox, Sigma), and 10 μM Rho-associated protein kinase (ROCK) inhibitor Y-27632 (RI, day 3). After 24 h, nascent neuritic extensions were visible under a microscope, the media was replaced with IM + dox but without RI (day 2) and the cells were incubated for 24 h. Fresh IM + dox was applied to the cells and they were returned to the incubator for a further 24 h (day 1). After 3 days of doxycycline treatment, neurites were clearly visible under a microscope. The day 0 i^3^N cells were cryo-preserved or plated onto Poly-L-ornithine coated cell culture vessels (for biochemistry, 1 × 10^5^ cells/cm^2^) or coverslips (for immunocytochemistry or electrophysiology, 5 × 10^4^ cells/cm^2^) in Cortical Media (CM); Brain Phys neuronal medium (STEMCELL Technologies), 1 × B27 supplement (Gibco), 10 ng/ml brain-derived neurotrophic factor (BDNF, Pepro Tech) in PBS containing 0.1% (w/v) IgG and protease-free BSA (Gibco), 10 g/ml NT-2 (Pepro Tech) in PBS containing 0.1% (w/v) IgG and protease-free BSA, 1 μg/mL laminin (Gibco). Half media changes every 4 days for the first week were followed by weekly half media changes.

### Immunocytochemistry

Characterisation of DIV 18 i^3^Ns was carried out by immunocytochemistry. Cells were plated on coverslips and fixed by incubation with 300 μL 4% (v/v) paraformaldehyde (PFA, Sigma) for 15 min at room temperature. PFA was aspirated and 300 μL PBS added to the well followed by blocking by incubation with 300 μL PBS containing 3% (v/v) Normal goat serum (NGS, Invitrogen) at room temperature for 1 h. Primary antibody, MAP2 (1:1,000) (Synaptic Systems 188004), was diluted in 350 μL 3% (v/v) NGS in PBS, which contained 0.1% (v/v) Triton-X (BDH) and incubated at 4°C overnight. Antibody was removed and cells were washed three times with a 10 min incubation of 350 μL PBS, 0.1% (v/v) Triton-X. Fluorescently labelled secondary antibody anti Guinea Pig^647^ (1:1,000) (Invitrogen A21450) was diluted in 300 μL 3% (v/v) NGS in PBS, 0.1% (v/v) Triton-X and incubated at room temperature for 1 h. Three PBS-Triton-X washes removed unbound antibody and PBS was added to each well. The plates were wrapped in foil and stored at 4°C until imaging on a Fluorescent Eclipse Ti2 microscope (Nikon) attached to an Intensilight C-HGF1 light source (Nikon) and a DS-Qi2 camera (Nikon) linked to a computer with NIS-Elements D imaging software (Nikon).

### Electrophysiology

Electrophysiological characterisation of DIV18 i^3^Ns was determined by single-cell electrophysiological (patch clamp) experiments to confirm that the i^3^N cell culture protocol yields electrically active neurons. Coverslips housing i^3^Ns were transferred to a recording chamber attached to a stage and upright microscope (Olympus BX50WI) equipped with infrared differential interference contrast optics for visualising individual cells. The cells were bathed in room temperature (24°C) artificial cerebrospinal fluid (aCSF, 124 mM NaCl, 3.2 mM KCl, 1.25 mM NaH_2_PO_4_, 26 mM NaHCO_3_, 2.5 mM CaCl_2_, 1.3 mM MgCl_2_, and 10 mM D-glucose) saturated with carbogen [95% (v/v) O_2_/5% (v/v) CO_2_], flowing at 2 ml/min for the duration of the experiments. Cells were patched with thick-walled borosilicate glass recording pipettes pulled on a Flaming/Brown micropipette puller (Sutter P-97) and filled with K^+^-based internal solution (135 mM K^+^ gluconate, 10 mM HEPES, 4 mM ATP-Na, 0.4 mM GTP-Na_2_, 10 mM phosphocreatine, 4 mM MgCl_2_). Recordings were made using a MultiClamp 700B amplifier coupled to a Digitizer 1440A A/D board and controlled using pClamp10 software (all hardware and software from Molecular Devices). Signals were low-pass filtered at 5 KHz and digitised at 20 KHz. All patch reagents were purchased from Sigma-Aldrich. ACSF salts/sugars were purchased from Merck.

Current clamp recordings were used to elicit action potentials (indicative of electrically active, mature neurons). Cells were held at resting membrane potential (∼−70 mV) and subjected to current steps of −100 pA for 200 ms in the presence or absence of the potent and selective Na^+^ channel blocker tetrodotoxin (TTX; 1 μM).

Voltage clamp recordings were obtained by clamping the neuronal membrane potential at −70 mV for a 5 min period to record spontaneous excitatory post synaptic currents (sEPSCs). The AMPA/Kainate receptor blocker NBQX (2,3-dioxo-6-nitro-7-sulfamoyl-benzo[f]quinoxaline, 10 μM) was washed in for the final minute of recording. Drugs were purchased from HelloBio ltd.

### Secreted Amyloid Precursor Protein Alpha Treatment and Time Course

At DIV18 sAPPα was added to the culture media to a final concentration of 1 nM, in a reverse time course, 2 h and 30 min (for transcriptome studies) and 24, 6, and 2 h (for proteome studies) prior to harvesting the cells. This concentration of sAPPα has been shown in many of our *in vitro* published studies to be the optimum concentration for eliciting its neurological effects (for example, Ryan et al., 2013). The 0 h control cells were not exposed to sAPPα and the reverse order time course ensured all cells had the same time in culture. Cell pellets were either frozen at −80°C until required for peptide generation for mass spectrometry or lysed directly in the culture vessel for immediate RNA extraction.

### Transcriptomics: RNA Sequencing

For the transcriptomics study, after media was aspirated and the cells rinsed with pre-warmed PBS, they were lysed directly in the wells of the plate by Buffer RL (350 μl) (Total RNA extraction kit, Norgen, Canada). Following 5 min incubation with swirling at room temperature the lysate was transferred to a microcentrifuge tube (2 ml), absolute ethanol added, and the solution mixed by vortexing. After centrifuging, supernatant was passed through a sterile 25-gauge needle 10× to shear the DNA, the RNA was bound to the Norgen column by centrifuging the solution for 1 min at 3,500 × *g* followed by three washes with Solution A of the kit (400 μl). Residual liquid was removed from the column by centrifuging for 2 min, and RNA was eluted with Elution Solution A (50 μl) into a fresh tube by centrifuging for 2 min at 200 × *g*, followed by 1 min at 14,000 × *g*. The RNA was stored at −80°C until use. It was treated with DNase-1 (ThermoFisher), purified (RNA clean up kit, Zymo Research) and the RNA integrity measured on a bioanalyzer (Agilent, United States). Very high-quality RNA (RIN > 9) was extracted from the human neurons by this procedure.

Nine samples of total RNA (0 h, 30 min, 2 h – each time point in triplicate) were submitted to the Otago Genomics Facility at the University of Otago (Dunedin, New Zealand) for library construction and sequencing. The libraries were prepared using TruSeq Stranded Total RNA Library Prep Gold kit (Illumina Inc., San Diego, CA, United States). All nine libraries were uniquely indexed and validated according to the manufacturer’s protocol. The nine pooled libraries at equimolar concentrations were then paired end sequenced across four lanes of HiSeq 2500 flow cells using V2 Rapid chemistry (Illumina), generating 100 bp reads. The FASTQ output files were trimmed [Trimmomatic (Bolger et al., 2014)], aligned [STAR alignReads function (Dobin et al., 2013)], and mapped to the G38 human genome (Genome Reference Consortium, National Centre for Biotechnology Information, NCBI). They were merged into one read count table for statistical analysis. Two pairwise comparisons were performed, which compared the 30 min data to the 0 h data, and the 2 h data to the 0 h data, to generate tables containing ratios (fold change) showing differential expression of reads for each genomic feature. The analysis was done using limma and edgeR. The voom function of limma was used to transform count data into logCPM (count per million), estimation of mean – variance relationship in the group and thus generating a table of weights. In the next step a linear model was fitted for each gene given the series of experiments (lmFit). Moderated *t*-statistics, moderated *F*-statistic, and log-odds of differential expression were computed by empirical Bayes moderation of the standard errors toward a common value (Ritchie et al., 2015). The 100 bp reads were aligned to the human genome and, after statistical stringency was applied, a set of differentially regulated transcripts was derived compared with no treatment with sAPPα (0 time). The three biological replicates are considered too low for false discovery rate to be applied (Schurch et al., 2016) resulting in removal of valuable data (false negatives), so the unadjusted *p*-values were used for the initial selection of candidates for further investigation. Significantly (*p* ≤ 0.05) differentially expressed (1.5-fold) transcripts were grouped, and common pathways and Gene Ontology (GO) terms were sorted using the STRING functional pathway analysis^1^ (version 11) and literature searching.

### Proteomics: Peptide Generation

For the proteomics study, the cells were first loosened from the plate by TrypLE™ Express Enzyme (Gibco, ThermoFisher) (500 μl) enzymatic digestion in each well of a 6-well plate. After rinsing with pre-warmed PBS, cells were harvested by centrifuging in an Eppendorf “lo bind” tube (1.5 ml), the pellets were snap frozen in an ethanol/dry ice bath and stored at −80°C until use.

For analysis, the frozen cell pellets from the four biological replicates of each sample at each time point were thawed in 200 μL digestion buffer [500 mM triethylammonium bicarbonate (TEAB)], 1 mM PMSF, 1 mM EDTA, 0.1% (w/v) SDS, 1% (w/v) sodium deoxycholate (SDC/DOC, Sigma), and homogenised with at least 20 grinds with a pestle creating shearing forces to break open the cells. The homogenate was vortexed for 10 s and sonicated for 1 min. After centrifuging at 16,000 × *g* for 30 min at 20°C the soluble fraction (supernatant) was retained, and contaminating DNA digested by addition of benzonase (100 U) (Sigma) followed by centrifuging at 16,000 × *g* for 30 min at 20°C. The supernatant was transferred to a 10 kDa molecular weight cut off centrifugal filter cassette unit (Amicon Ultracel – EMD Millipore), carefully avoiding the top lipid layer and the bottom pellet (DNA). Samples were further processed following the protocol for filter-aided sample processing (FASP) (Wiśniewski, 2017). In brief detergents (SDS and DOC) were depleted by washing the samples in 8 M urea in 200 mM TEAB followed by reduction and alkylation of disulphide bonds in 5 mM TCEP [Tris(2-carboxyethyl)phosphine] in 200 mM TEAB and 10 mM iodoacetamide in 200 mM TEAB, respectively. Reduced and alkylated proteins were recovered from the cassette by inversion of the filter unit placed onto a new 1.6 mL microcentrifuge tube and centrifuging. To ensure complete recovery a further 50 mL TEAB buffer was added to the filter cassette, vortex mixed, and inverted to collect the remaining protein solution by centrifugation.

The protein content of each sample was quantified with the Bradford protein quantitation assay (Bio-Rad protein assay, Bio-Rad). Peptides were generated by digesting 100 μg protein with 5 μg Trypsin (1/20th trypsin) gently vortexed and incubated overnight at 37°C. A “tryptic boost” with half the amount (2.5 μg) of the trypsin used for the overnight incubation, was added to the protein/peptide solution, gently vortexed and incubated for a further 5 h at 37°C. Peptides were purified and concentrated by Solid Phase Extraction (SPE) on Sep-Pac C18 cartridges (Waters).

### Quantitative Proteomics

Proteins were identified and quantified by SWATH-MS as described in detail elsewhere (Sweetman et al., 2020). In brief an aliquot of each sample was pooled into a reference sample that was used to generate a comprehensive spectral library. The peptides of this complex reference sample were pre-fractionated into 11 fractions by high pH reverse phased fractionation on C18 cartridges (Pierce™ High pH Reversed-Phase Peptide Fractionation Kit, Thermo Fischer Scientific) according to the manufacturer’s protocol. Each fraction was then analysed in technical duplicates by data-dependent acquisition (DDA) mass spectrometry for protein identification using a 5600 + Triple Time-Of-Flight (TOF) mass spectrometer coupled to an Eksigent “ekspert nanoLC 415” uHPLC system (AB Sciex). For peptide quantification each individual sample was subjected to data-independent acquisition (DIA) using SWATH-MS in three technical replicates. For SWATH-MS the same instrument setup and LC-gradient settings were used as used for DDA-MS to allow an accurate alignment of the DIA peak intensities to the spectral library.

Data-dependent acquisition raw data was searched against the human reference sequence database (87,570 sequence entries, downloaded from the NCBI server^2^ on 29/03/2019) using the ProteinPilot software version 4.5 (AB SCIEX). Significant peptide identifications at a false discovery rate (FDR) of ≤1% and a confidence of ≥95% were loaded into the SWATH Acquisition MicroApp 2.0 of the PeakView software (version 2.2, ABSciex) to build a spectral library. The spectral information from SWATH-MS raw data was then matched to library spectra using a time window of 12 min and a mass accuracy of 50 ppm. The intensities of the 6 strongest fragment ions from each of the 10 strongest peptides per protein that were matching the library spectra at a FDR ≤ 1% were imported into the MarkerView software (version 1.2, AB Sciex) for quantification. Intensities were normalised between the different sample runs based on the total sum of peak intensities. Unsupervised multivariate statistical analysis using principal component analysis (PCA) was then performed in the MarkerView software for sample grouping and comparison. A *t*-test of the median value of the technical replicates was carried out comparing the sAPPα group with the PBS control group, generating a dataset of proteins with significantly different relative abundances between the two groups (*p* ≤ 0.01, minimum 1.5-fold change). Any proteins with a coefficient of variance over 40% of their abundance between the technical replicates were also removed from the data set. The significantly differentially expressed proteins were investigated by searching the literature and by using the STRING database (see text footnote 1) to identify functional association networks and identify potential interactions. The STRING analysis considered the differentially expressed protein lists against the three species commonly used in sAPPα research (mouse, rat databases as well as human) as some data was more enriched in the mouse and rat, and this resulted in a wider array of GO term enrichment outputs, some highly relevant to neurological function.

## Results

### Secreted Amyloid Precursor Protein Alpha Production

The protein produced after expression in HEK cells in culture was highly purified to homogeneity and was identified as sAPPα by Western blotting (Figure 1B lane 2 – SDS polyacrylamide gel stained with Coomassie blue, and lane 3 – Western blot with an N-terminal APP antibody). The protein was larger than that theoretically derived from the amino acid sequence (72 kDa) as there are known post translational glycosylations added to the protein in human cells. The glycosylated protein has a molecular weight of ∼100 kDa. This preparation was used to determine the effect of sAPPα on the human neuron cells’ transcriptome and proteome.

### i^3^N Cell Culture Characterisation

Isogenic cell cultures of human i^3^Ns were derived from a doxycycline iPSC cell line that had been stably integrated with the neuron transcription factor neurogenin-2 (Wang et al., 2017; Fernandopulle et al., 2018). After 18 days *in vitro* these cells as shown below exhibited electrophysiological properties of glutamatergic neurons and expressed neuronal markers specific to the pre- and post-synapse, axon, and dendrites and have a morphology that is typically neuronal with long neurites projecting from a cell body.

Brightfield microscopy shows the differentiation of iPSC’s into neurons (Figure 1C). The iPSCs grow in tight isolated colonies (day 5) but are dissociated to a single cell suspension prior to the induction of differentiation by addition of doxycycline to the culture media (day 3, not shown). The differentiating cells begin to elongate and no longer grow in colonies (day 1). Neurites (axons and dendrites) grow out from the soma (day 1) which lengthen (day 6) and form connections with other cells (day 13).

Immunocytochemistry experiments confirmed that the i^3^Ns expressed neural markers for dendrites (MAP2) (Figure 1C). Previous studies from the group have shown using confocal microscopy the post synaptic protein (homer), pre-synaptic proteins (synaptophysin) and the axon marker (tau) in these cells (Basak et al., 2021). For the studies here characterisation was repeated using fluorescence microscopy and confirmed the presence of these markers

### Electrophysiology Characterisation

**FIGURE 2:**
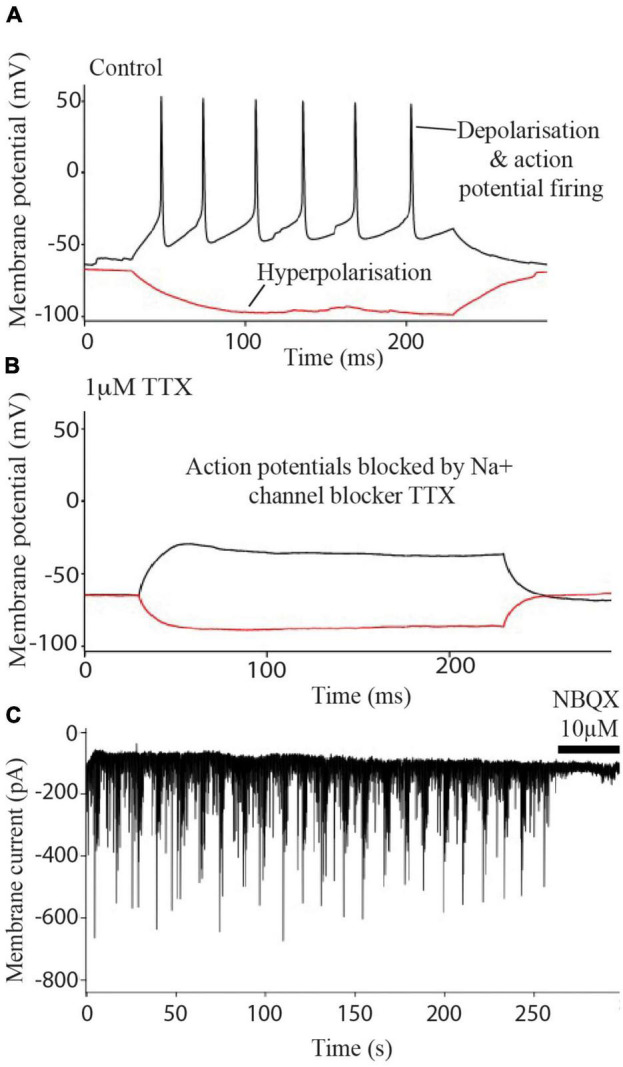
Electrophysiology of iPSC derived neurons. Neurons fire action potentials **(A)** which are blocked after the addition of 1 μm TTX **(B)**. Excitatory post synaptic currents **(C)** are abolished after treatment with 10 μM NBQX.

Experiments determined that the i^3^N were typical electrically active neurons firing Na^+^ channel-dependent action potentials (Figure 2A) which were blocked after treatment with the sodium channel blocker TTX (Figure 2B). Blockade of sEPSCs by NBQX (Figure 2C) indicated that the currents were due to AMPA-type glutamate receptor activation i.e., these neurons form glutamatergic synapses.

### RNA Sequencing

**FIGURE 3:**
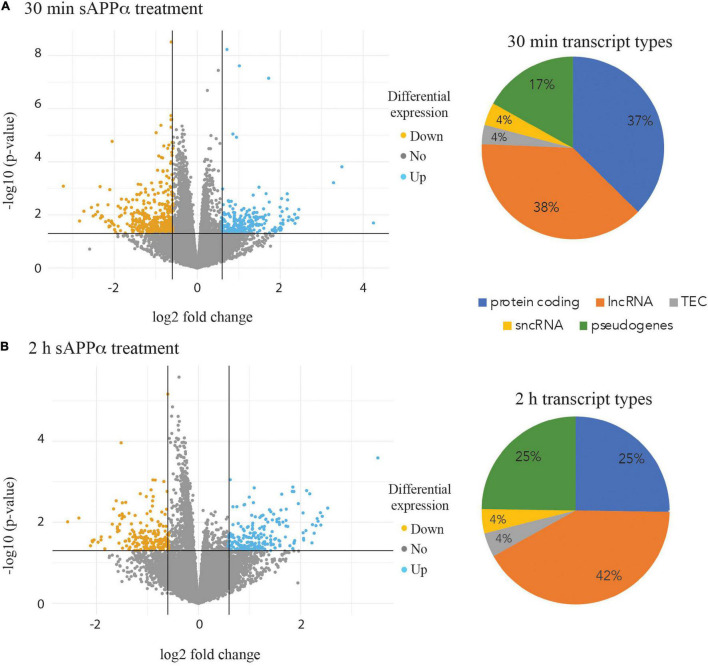
Volcano plots highlighting differential expression of individual transcripts. Transcripts down regulated (orange points) and up regulated (blue points) are shown after 30 min **(A)** and 2 h incubation of the human neurons with 1 nM sAPPα **(B)**. Pie charts illustrating the proportion of biotypes within the group of significantly differentially expressed transcript groups in the 30 min **(A)** and 2 h **(B)** data. lncRNA – long non coding RNA, sncRNA – short non coding RNA, TEC – transcript type to be experimentally confirmed.

The isolated RNA from three biological replicates at each of the three time points (0, 30 min and 2 h) after incubation with 1 nM sAPPα was subjected to RNA sequencing. A total of 25,272 expressed transcripts were identified in the i^3^N cells. Pairwise comparisons were made between both the 30 min and 2 h data against the 0 h time point data. Differentially expressed transcripts were selected if the (log 2) fold change was ±0.58 or with a *p*-value of ≤0.05. After 30 min exposure to exogenous sAPPα 645 differentially expressed transcripts were identified (Figure 3A), and 408 were identified after 2 h of exposure (Figure 3B). Biotype analysis of these transcripts revealed that 37% (30 min) and 25% (2 h) were protein coding genes, 38% (30 min) and 42% (2 h) were lncRNAs and 17% (30 min) and 25% (2 h) pseudogenes. The full lists of differentially expressed gene transcripts and their biotype at each time point are shown in Supplementary Table 1.

There were 241 differentially expressed protein coding transcripts at 30 min and 103 at 2 h. Differentially expressed protein coding genes from each time point were subjected to STRING functional network analysis. Table 1 shows the GO terms for enriched biological process, molecular function, cellular component and Uniprot Keywords within the 30 min data.

**TABLE 1:** Enrichment of GO terms in RNA sequencing data.

RNA sequencing data 30 min					
**# Term ID**	**Term description**	**Observed gene count**	**Background gene count**	**Strength**	**False discovery rate**
**Molecular function enriched GO terms**					
GO:0005109	Frizzled binding	6	39	1.11	0.0154
GO:0017147	Wnt-protein binding	5	33	1.11	0.045
GO:0001664	G protein-coupled receptor binding	16	294	0.66	0.0044
GO:0030545	Receptor regulator activity	20	536	0.5	0.0154
GO:0048018	Receptor ligand activity	18	490	0.49	0.0252
**Biological Process enriched GO terms**					
GO:0060070	Canonical wnt signalling pathway	8	85	0.9	0.0341
GO:0009887	Animal organ morphogenesis	29	967	0.4	0.0193
GO:0007267	Cell-cell signalling	33	1,145	0.39	0.0128
**Cellular Component enriched GO term**					
GO:0005886	Plasma membrane	94	5,314	0.17	0.0206
**Uniprot Keywords**					
KW-0879	Wnt signalling pathway	12	194	0.72	0.0028
KW-0272	Extracellular matrix	12	265	0.58	0.0255
KW-0964	Secreted	48	1,818	0.35	0.00013

The protein coding transcripts differentially expressed at 30 min were grouped by their association with GO Pathway categories, following a STRING functional analysis.

The protein coding transcripts differentially expressed at 30 min were associated with the following GO Pathway categories.

(i) GO **Biological Functional** categories linked the differentially expressed transcripts to specific neuronal roles, including neuronal fate determination and WNT signalling. Cell signalling was a predominant class that included cell–cell signalling, cell surface signalling pathways, morphogenesis, cell communication, regulation in response to signalling, signal transduction, and calcium independent cell adhesion *via* plasma membrane adhesion molecules. (ii) GO **Molecular Function** categories included WNT binding, G protein coupled receptor binding, DNA-binding transcription activator activity, and signalling receptor binding, while (iii) GO **Cellular Component** terms indicated a large proportion of proteins affected by sAPPα were localised to the plasma membrane, cell periphery or extracellular space.

**FIGURE 4:**
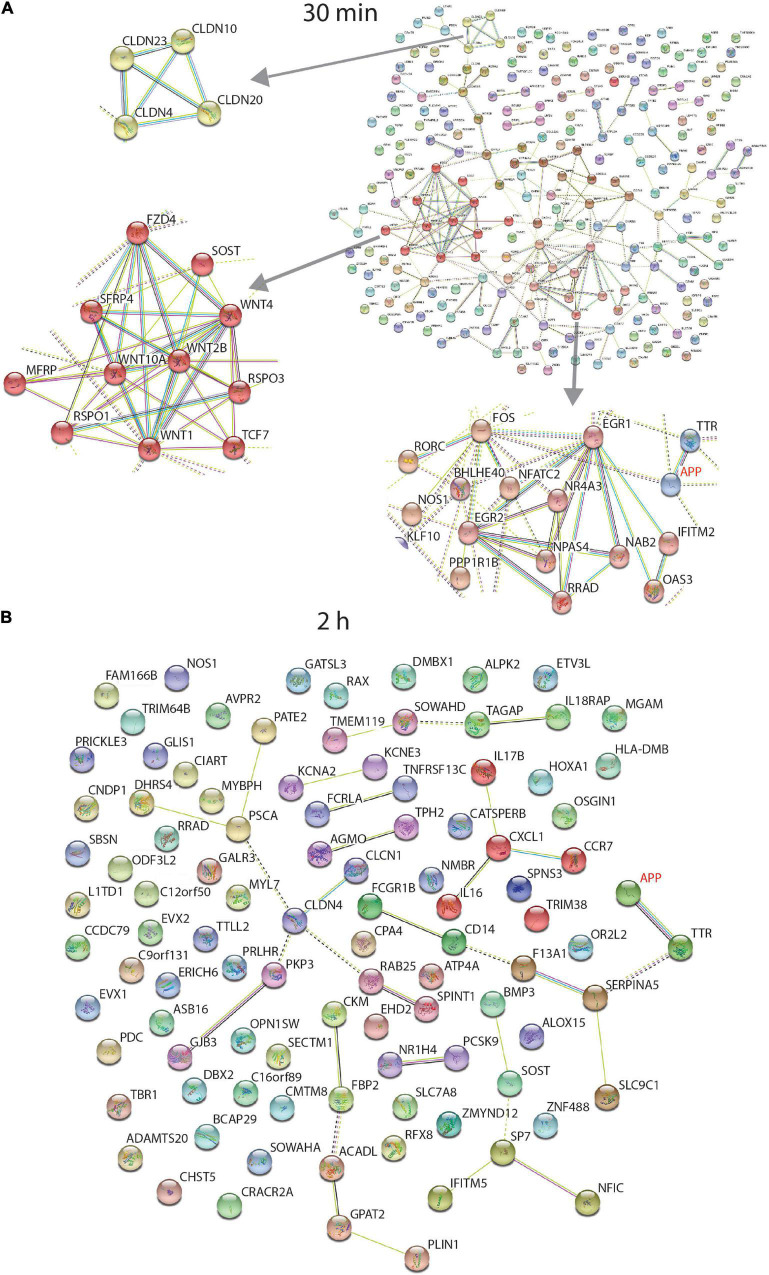
STRING interaction network of differentially expressed protein coding genes. Data from RNA sequencing at 30 min **(A)** and 2 h **(B)**. At 30 min, incubation of the human neurons with 1 nmM sAPPα, differentially expressed transcripts were associated with enriched GO terms (coloured). There were 12 extracellular matrix proteins (purple), 48 secreted proteins (red) and 94 proteins associated with the plasma membrane (green). Three individual clusters shown at left were identified by performing MCL clustering in the 30 min data which included a cluster of Claudin (CLDN), WNT signalling and Early Response genes. The 2 h data had no enriched GO terms. Coloured proteins (nodes) are coloured representing different clusters identified after applying the Markov Clustering Algorithm (MCL) (Brohée and van Helden, 2006).

Figure 4 shows the STRING functional network diagram for protein coding genes identified as differentially expressed after 30 min and 2 h. Three individual clusters were identified by performing MCL clustering in the 30 min data (Figure 4A) which included a cluster of Claudin (CLDN), WNT signalling and Early Response genes.

No GO term enrichment was found after a STRING analysis of the 2 h data (Figure 4B).

A literature search of the protein coding transcripts revealed that ∼30% from both 30 min and 2 h data were involved with cell signalling, neurotransmitters, receptors and ion channels, gene transcription, actin and tubulin dynamics, ECM and cell adhesion. Additionally, the 30 min data contained eight early response genes/immediate early genes (EGR1, EGR2, FOS, KLF10, E2F8, NPAS4, BAZ2A, and CCN1).

Table 2 lists the protein encoding genes in more detail under the subheadings of (i) *early response genes and transcriptional regulators*, (ii) *cell signalling*, (iii) *neurotransmitters, receptors, and channels*, (iv) *actin and tubulin dynamics*, (v) *extracellular matrix and cell adhesion*, and (vi) *APP related proteins.* This data set indicates that sAPPα by 30 min rapidly initiates an extensive new gene expression network for the human neuron cell in culture.

**TABLE 2:** RNA sequencing data selection of protein coding genes differentially expressed after 30 min incubation with 1 nM sAPPα and their functions.

Protein coding transcripts differentially expressed at 30 min					
Gene	Reg.	References	Gene	Reg.	References
**Early response genes and transcriptional regulators**			**Receptors, channels, and transporters**		
*EGR1*	Up	Duclot and Kabbaj, 2017	*ARRDC4*	Down	Foot et al., 2017
*EGR2*	Up	Poirier et al., 2007	*SLC7A8*	Down	Albrecht and Zielińska, 2019
*FOS*	Up	Gandolfi et al., 2017	*SLC10A1*	Up	Ayka and Şehirli, 2020
*KLF10*	Down	Subramaniam et al., 2010	*SLC16A6*	Down	
*E2F8*	Down	Weijts et al., 2012	*SLC16A9*	Down	
*NPAS4*	Up	Sun et al., 2020	*SLC9C1*	Up	
*BAZ2A*	Down	Guetg et al., 2010	*NOS1*	Down	Paul and Ekambaram, 2011
*NKX2-2*	Down	Jarrar et al., 2015	*CACNG5*	Down	Milstein and Nicoll, 2008
**Cell signalling**			*ATP12A*	Down	Larsen et al., 2016
*SPRY2*	Up	Impagnatiello et al., 2001	*ATP4B*	Down	
*WNT4*	Down	Inaki et al., 2007	*HTR2B*	Up	Wood et al., 2011
*WNT1*	Down	Budnik and Salinas, 2011	*CLCN1*	Down	Chen et al., 2013
*WNT10A*	Down		*OTOF*	Down	Takago et al., 2018
*WNT2B*	Down		**Extracellular matrix and cell adhesion**		
*RSPO1*	Down	Jin and Yoon, 2012	*ADAMTS8*	Down	Gottschall and Howell, 2015
*RSPO3*	Down		*ADAMTS15*	Down	
*S100A11*	Down	Zhang et al., 2021	*NECTIN1*	Down	Mizoguchi et al., 2002
*PLEKHA4*	Down	Lenoir et al., 2015	*CLDN10*	Down	Tikiyani and Babu, 2019
*PLEKHG6*	Down	O’Neill et al., 2018	*CLDN20/23*	Down	
**Actin and microtubule dynamics**			*CLDN4*	Down	
*ADM*	Up	Larrayoz et al., 2017	*PCDHGA6*	Down	Weiner et al., 2005
*CHAMP1*	Down	Hempel et al., 2015	*PCDHGB2*	Down	
*CAPN6*	Down	Chen et al., 2020a	*CCN1*	Up	Chen and Lau, 2009
*LIMS2*	Down	Xu et al., 2016	**APP and AD related**		
*DARPP-32*	Up	Svenningsson et al., 2004	*SERPINC1*	Down	Sanrattana et al., 2019
–	–	–	*SERPINE1*	Down	Ho et al., 1994
–	–	–	*TTR*	Down	Li and Buxbaum, 2011
–	–	–	*CXCL1*	Up	Zhang et al., 2015

Immediate early genes (IEGs) involved in memory formation were differentially expressed after 30 min and represent one of the clusters in Figure 4A. Early growth factor 1 (EGR 1, Zif268, Krox24) is upregulated 3.3-fold after 30 min. SNAP29 and PSD-95 are genes modulated by EGR1 and these were identified as differentially expressed in the proteome data after 24 h (Duclot and Kabbaj, 2017). A transcriptional repressor (NAB2) for EGR1 is downregulated 1.5-fold, which is consistent with the increase in EGR1 and 2 (Russo et al., 1995). Upregulated IEGs FOS and NPAS4 encode two different types of memory engrams from the same experience (Sun et al., 2020). APP promotor contains an AP1 binding site required for FOS binding potentially modulating APP transcription (Trejo et al., 1994).

WNT signalling related molecules were enriched in these data and from another cluster in Figure 4A. WNTs are involved during synaptic development where their expression is increased after depolarisation and NMDA receptor activation in the hippocampus, leading to dendrite development (Budnik and Salinas, 2011).

Five solute carriers were differentially expressed. These membrane proteins transport solutes across membranes and their dysregulation is implicated in neurodegenerative disease (Ayka and Şehirli, 2020). One of these, the glutamine transporter SLC7A8, is downregulated, which may impact on levels of glutamate in the neuron (Albrecht and Zielińska, 2019). Stimulation of NMDA receptors by glutamate triggers the release of Nitric Oxide, synthesised by NOS, which is downregulated here 2.6-fold. NO acts as a retrograde messenger for the induction of LTP and LTD in the hippocampus (Zorumski and Izumi, 1998). Trafficking and channel gating of AMPA receptors is regulated by TARPs like CACNG5 which is downregulated (Milstein and Nicoll, 2008).

The proadrenomedullin gene (upregulated here) generates two neuromodulatory peptides (i) AM which binds to microtubule associated proteins (MAPs) and (ii) PAMP which binds to kinesin increasing the transportation of cargo (Larráyoz and Martínez, 2012). Ablation of the ADM gene which is increased in aging mice, improves memory performance (Larrayoz et al., 2017). DARPP-32 is concentrated in dendritic spines (Blom et al., 2013) where it functions as a kinase or phosphatase inhibitor after stimulation by glutamate or dopamine (Svenningsson et al., 2004).

ADAM proteins are extracellular proteases which maintain the perinuclear net (ECM) environment required for synaptic connectivity and their dysregulation causes neurodegeneration (Gottschall and Howell, 2015). Two ADAMs are downregulated here.

Cell adhesion molecule NECTIN1 is involved with the formation of adhesive sites between cells at the synapse (Mizoguchi et al., 2002). A decrease (as seen here) in NECTIN1 level, increases the number of synapses (Honda et al., 2006). Another group of cell adhesion molecules are Claudins (CLDN). They also form a network cluster in Figure 4A. They form tight junctions controlling the permeability of the membrane to water and ion, Claudin-like proteins interact with actin and actin binding proteins at the synapse (Tikiyani and Babu, 2019) and Claudin family member Stargazin acts as an obligate auxiliary subunit of transmembrane AMPA receptors that is required for glutamatergic currents (Walker et al., 2006; Wang et al., 2008). Procadherin (PCDH) family members (downregulated) are neural cadherin-like cell adhesion proteins detected in synapses and at growth cones (Weiner et al., 2005).

In summary, by 30 min of sAPPα exposure there is a differential expression of IEG’s and WNT signalling molecules that will have effect on downstream genes, and genes that facilitate transport of solutes and remodelling of cellular structures like the synapse.

For the 2 h time point by contrast not only were there fewer differentially expressed protein transcripts but there was no overrepresentation of GO pathways. Nevertheless, 30 differentially expressed transcripts were common to both time points and for those downregulated this was reduced at 2 h in all cases. For the upregulated genes three genes had further enhanced upregulation at 2 h but the remainder were also reduced.

Table 3 shows a list of those differentially expressed genes at 2 h with links to neuronal function or APP, they separated into the following categories (i) *receptors, channels, and transporters*, (ii) *cell signalling*, (iii) *actin and microtubule dynamics*, and (iv) *extracellular matrix and cell adhesion*.

**TABLE 3:** RNA sequencing data selection of protein coding genes differentially expressed after 2 h incubation with 1 nM sAPPα and their functions.

Protein coding transcripts differentially expressed at 2 h					
Gene	Reg.	References	Gene	Reg.	References
**Receptors, channels, and transporters**			**Cell signalling**		
*SLC7A8*	Down	Albrecht and Zielińska, 2019	*SBSN*	Up	Pribyl et al., 2021
*SLC9C1*	Up	Ayka and Şehirli, 2020	*IL16*	Down	Fenster et al., 2010
*NOS1*	Down	Paul and Ekambaram, 2011	*IL17B*	Down	Moore et al., 2002
*KCNA2*	Down	Hyun et al., 2015	**Extracellular matrix and cell adhesion**		
*KCNE3*	Up	McCrossan et al., 2003	*CLCN1*	Down	Tikiyani and Babu, 2019
**Actin and microtubule dynamics**			*CLDN4*	Down	
*MYBPH*	Down	Hirokawa et al., 2010	*GJB3*	Down	Xia et al., 1998
*CXCL1*	Up	Zhang et al., 2015	*ADAMTS20*	Up	Kurshan et al., 2014

Some notable protein coding genes at 2 h include *ADAMTS20*, *KCNA2*, *ABSN*, and *IL17B* which may have important neural functions. In Caenorhabditis elegans gon-1, a conserved extracellular ADAMTS protease (Homologous to ADAMTS20 shown upregulated here), is required for maintaining proper synaptic morphology at the neuromuscular junction (Kurshan et al., 2014). KCNA2 (Kv1.2) is a potassium voltage gated channel involved in the induction of LTP (Hyun et al., 2015) and KCNE3 is also known as MinK-related peptide 2 which modulates potassium channels in the brain to determine firing frequency (McCrossan et al., 2003).

SBSN (Suprabasin) is involved with WNT, AKT and P38MAPK signalling (Pribyl et al., 2021) and induces sprouting during angiogenesis (Takahashi et al., 2020). Interleukins are a family of cytokines, IL16 (downregulated) induces c-fos expression and neurite outgrowth (Fenster et al., 2010). IL17B (down regulated) protein is localised to neuronal cell bodies and is linked to Charcot–Marie–Tooth (CMT) demyelinating disease (Moore et al., 2002). Another gene linked to CMT and deafness is the connexin, GJB3 (downregulated) which is a gap junction protein (Xia et al., 1998). Gap junctions are specialised cell–cell contacts that provide direct intracellular communication. They allow passive diffusion of molecules up to 1 kDa, including nutrients, metabolites (glucose), ions (K^+^, Ca^2+^) and second messengers (IP3, cAMP).

### Sequential Window Acquisition of All THeoretical Fragment Ion Spectra-Mass Spectrometry

For the proteome study human iPSCs were first differentiated into glutamatergic neurons (i^3^N’s) as described above. Mature i^3^N’s were treated with 1 nM sAPPα with four biological replicates in a reverse time course for 24, 6, 2 h, or harvested immediately (0 h) so all cells had been in culture for the same time. A comparison of the differentially regulated proteins at each time point compared with the no sAPPα 0 h time control reflects the dynamic changes in protein expression and abundance that sAPPα is mediating. Cell pellets were processed to isolate proteins and generate peptides. Firstly, shotgun mass spectrometry proteomics of the pooled reference sample significantly identified 4,500 proteins, which were integrated into the spectral library. After matching the SWATH-MS spectral data against the library, 3,233 proteins were quantified at a sufficient intensity and low variability among the three technical replicates.

**FIGURE 5:**
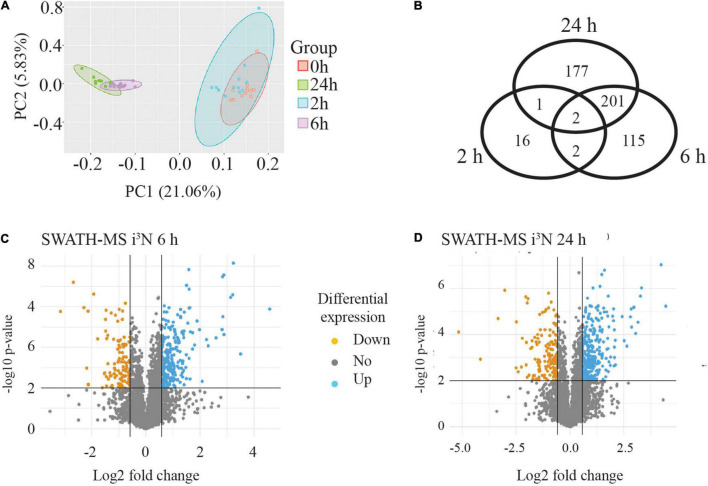
SWATH-MS data analysis from human neurons treated with sAPPα. **(A)** PCA analysis illustrates four biological replicates and three technical replicates of the samples for each time point. The data from each time point is within an elipse. It showed that the data from 6 and 24 h data segregated from the 0 and 2 h data. **(B)** Venn diagram of significantly (*p* ≤ 0.01) differentially regulated proteins revealed the number of proteins exclusive and in common to each time point compared to the zero time point. Inverse volcano plots the −log10 *p*-value (*y*-axis) against Log2 fold change (*x*-axis) at 6 h **(C)** and 24 h **(D)** to highlight any down regulated (orange point) and up regulated (blue point) proteins which meet criteria for selection of a minimum 1.5-fold change (log2 fold change ≤ −0.58 or ≥ 0.58) with *p*-value ≤ 0.01.

A PCA was carried out to assess variability in the data across all the four time points and among the four biological replicates of each time point. This showed that the four biological replicates from each time point (separately coloured) clustered together and were tightly grouped with their technical replicates. The PCA analysis showed that the biological replicates of the 0 and 2 h time points tended to group together but were clearly separated from the 6 and 24 h samples, which were themselves clustered together. This is illustrated in Figure 5A where the ellipses were drawn around the data sets for each time point to see how the data grouped together (four biological replicates and three technical replicates for each time point). A Venn diagram of the differentially expressed proteins (minimum 1.5-fold change, statistical stringency, *p* ≤ 0.01) when compared to the untreated sample (0 h) at each time point is shown in Figure 5B. At 2 h, of the 21 differentially expressed proteins, 16 were differentially expressed at this time point only, and 2 were found at all time points compared with the untreated 0 h condition. At 6 h there were 115 proteins unique to this time point, and 201 shared with 24 h only, and only 2 shared with 2 h, and 2 with the other time points. There were 177 proteins uniquely differentially expressed at the 24 h time point

The median value of the technical replicates for each protein at each time point was taken to form ratio changes of 2 or 6 or 24 vs 0 h for each protein, and this is also referred to as the fold change measurement. The fold change values were converted to their log2 equivalent. A student two-tailed *t*-test was performed on the data to predict the significance (*p*-value) of the observed changes. Volcano plots were generated by plotting the −log10 *p*-value (*y*-axis) against log2 fold change (FC, *x*-axis) for each protein. The volcano plots show proteins as individual points, coloured in orange or blue if they are significantly down- or upregulated, respectively (Figures 5C,D). Differentially regulated proteins were initially considered for analysis if they reached a threshold of log2(FC) ± 0.58 (1.5-fold change) with a *p*-value ≤ 0.01.

For the 2 h time point there were only 11 upregulated and 8 down regulated proteins identified. These data are shown in Supplementary Table 2. By contrast, for the 6 vs 0 h data, there were many more differentially regulated proteins at the stringency of *p* ≤ 0.01 (320, with 222 upregulated and 98 downregulated) (Figure 5C), and also for the 24 vs 0 h data, where there were 380 proteins identified, 265 differentially upregulated and 115 downregulated (Figure 5D).

The full lists of differentially regulated proteins are shown in Supplementary Table 2 for each time point.

**FIGURE 6:**
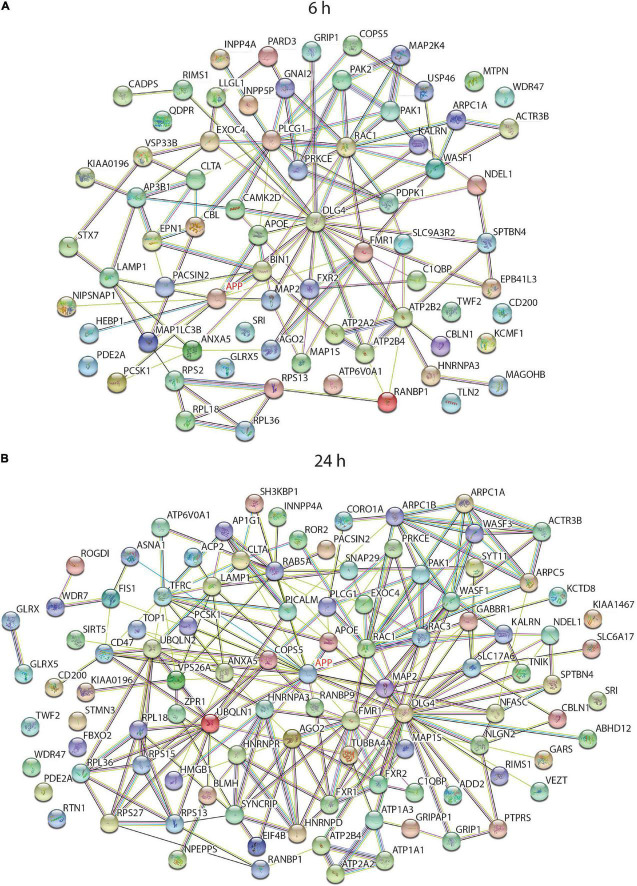
String analysis of functional interactions of differentially regulated proteins after incubation of neurons with sAPPα. Diagram shows the significantly differentially regulated proteins, which were linked to cellular component GO terms with a neural functions and/or had a connection to APP, and their relationship to others in the 6 h **(A)** and 24 h **(B)** data set when searched against the human protein database (log2 (FC) -/+ 0.58, p ≤ 0.01).

To find any common biological pathways or networks in which these proteins may be involved, a STRING functional network analysis was performed on the differentially expressed proteins from each time point and some of the enriched pathways are highlighted (Figure 6). As APP is the protein from which sAPPα is derived it was of interest to find any proteins in the data sets which had links to APP. In each case APP was added to the STRING search to determine if there are any known relationships between it and any of the differentially expressed proteins. Since sAPPα is known to influence learning and memory mechanisms, and the structure in the cell responsible for this ability is primarily the synapse, any proteins in the data set that were associated with the neuronal projections (dendrites and axons) and the synaptic regions have been highlighted (Figures 6A,B).

The STRING algorithm calculates when there are enough proteins belonging to a group to be considered enriched. For example, there were 47 proteins in the neuron projection category at 24 h, but at 6 h this category was not considered enriched. A manual analysis however, compared the protein lists back to the 6 h data and noted several relevant proteins occurring in that data set as well. Supplementary tables contain the comprehensive list of enriched GO terms for 6 h (Supplementary Table 3) and 24 h (Supplementary Table 4) time point.

As illustrated in the string diagrams for the 6 h (Supplementary Figure 1) and 24 h (Supplementary Figure 2) time points, for all differentially expressed proteins there were many functional interactions within these complex patterns. Of the 320 differentially expressed proteins at 6 h (Supplementary Figure 1), over 80% belonged to one or more of the gene-enriched ontology groups. Of those related to neuronal functions, 15 proteins had an association with APP. Cellular compartment enrichment included many proteins that were associated with the GO terms “synapse” (42), “growth cone” (11), vesicle (112), mitochondria (65), and cytoskeleton (22). APOE and DLE4 were involved with the enriched biological process GO term “AMPA receptor clustering,” and three proteins with “axon hillock.” When the list of proteins was searched against the mouse database the “Axon guidance reactome pathway” was enriched with nine differentially expressed proteins. In the summary of the analysis data from the 24 h time point linked to neurological function (Supplementary Figure 2), APP was now linked with 20 different proteins, and GO Cellular Component terms enriched included “synapse, neuron projection, spliceosomal complex, cell junction, mitochondrial, and actin cytoskeleton.” GO biological processes had highly significant categories of “modulation of excitatory post synaptic potential,” “membrane biogenesis, regulation of exocytosis, regulation of actin filament polymerisation, and regulation of RNA splicing.”

To tease out functionally important interactions with APP, a STRING diagram associated with “Neuronal projection” and “Synapse” GO terms were generated to observe how they interacted with each other and the experimental effector protein sAPPα (as APP). This is illustrated in Figures 6A,B (the full list of proteins associated with each GO term is in Supplementary Table 5). This clearly illustrates that sAPPα can change the overall gene expression of the human neuron for functions critical to its role as a neural cell not only directly but also indirectly as it spreads out to affect many other cellular and biological functions.

The full lists of the differentially expressed proteins are shown in Supplementary Table 2 and those proteins highlighted as having neuronal function or linked to APP or AD are shown in Supplementary Table 6 (6, 24 h). Those related to APP included one differentially regulated protein at 2 h, 9 proteins at 6 h and 21 proteins at 24 h after sAPPα application. Of previously identified Alzheimer’s risk genes (Rosenthal et al., 2012), four proteins encoded by them were differentially expressed, for example, APOE was upregulated at 6 and 24 h, and PICALM was downregulated at 24 h, BIN1 upregulated at 6 h, and SH3KBP1 was upregulated at 6 and 24 h. These are listed in more detail in Table 4 (6 h) and Table 5 (24 h). The references in the table describe the protein in question. Categories included in Table 4 are (i) *synaptic vesicle*, (ii) *APP and AD related*, (iii) *neurite outgrowth and synapse*, (iv) *actin and microtubule dynamics*, (v) *neurotransmitters receptors and ion channels*, and (vi) *lysosome and proteasome*. Hence, the proteins discussed here Table 4 (6 h) relate to GO terms with (i) neuronal functions or (ii) have known association with APP (as predicted by the STRING network analysis) or (iii) are associated with a neurological disorder.

**TABLE 4:** SWATH-MS data selection of proteins differentially regulated after 6 h exposure to sAPPα.

Proteins differentially regulated after 6 h					
Protein	Reg.	References	Protein	Reg.	References
**Synaptic vesicle**			**APP and AD related**		
CLTA	Up	Redlingshöfer et al., 2020	APOE	Up	Saunders et al., 1993
BIN1	Up	De Rossi et al., 2020	CD200	Up	Varnum et al., 2015
NIPSNAP1	Down	Antonucci et al., 2016	COPS5	Up	Wang et al., 2013
RIMS1	Up	Lonart, 2002	PCSK1	Up	Maftei et al., 2019
VAPA	Up	Lindhout et al., 2019	**Actin and microtubule dynamics**		
EPN1	Down	Jakobsson et al., 2008	MAP2	Up	Johnson and Jope, 1992
SNX12	Up	Zhang et al., 2018	MAP1S	Down	Bodakuntla et al., 2019
SNX2	Up		MAP1LC3B2	Up	
STX7	Up	Mori et al., 2021	MAP2K4	Up	
EXOC4	Down	Liebl et al., 2005	MAPRE3	Up	
**Neurite outgrowth and synapse**			WDR47	Up	Chen et al., 2020c
CBLN1	Up	Yuzaki, 2011	CORO2A	Up	Marshall et al., 2009
PSD95	Down	Coley and Gao, 2018	CORO2B	Down	Chen et al., 2020b
TWF2	Up	Yamada et al., 2007	PACSIN2	Up	Ritter et al., 1999
SPTBN4	Down	Machnicka et al., 2014	MTA2	Down	Covington and Fuqua, 2014
WASHC5	Down	Freeman et al., 2013	ACTR3B	Down	Galloni et al., 2021
RABIF	Down	Gulbranson et al., 2017	AP3B1	Up	Li et al., 2016
RAC1	Up	Haditsch et al., 2009	ARPC1A	Up	Spence et al., 2016
RALA	Up	Li et al., 2007	KALRN	Up	Mandela and Ma, 2012
RALB	Up		PFDN1	Down	Liang et al., 2020
RANBP1	Up	Mignogna and D’Adamo, 2018	PFDN4	Up	
RANBP3	Down		PFDN6	Up	Gu et al., 2008
NDEL1	Down	Kuijpers et al., 2016	TLN2	Down	Morgan et al., 2004
AGO2	Up	Rajgor et al., 2018	**Receptors, channels, and transporters**		
IGFBPL1	Up	Guo et al., 2018	GRIP1	Up	Tan et al., 2015
CADM4	Up	Tanabe et al., 2013	INPP4A	Down	Sasaki et al., 2010
**Lysosome and proteasome**			SLC25A22	Up	Hu et al., 2020
USP10	up	Lim et al., 2019	SLC41A3	Down	
USP15	down	Young et al., 2019	SLC9A3R2	Up	
USP24	up		FXR2	Up	Guo et al., 2015
USP46	up		FMR1	Up	Telias, 2019
CBL	up	Zhang et al., 2020	ATP2A2	Up	Mata and Sepulveda, 2010; Larsen et al., 2016
KCMF1	down	Hong et al., 2015	ATP2B2	Down	
UBE3A	down	Vatsa and Jana, 2018	ATP2B4	Up	
–	–	–	ATP6V0A1	Up	
–	–	–	LAMP1	Up	Hwang et al., 2019

**TABLE 5:** SWATH-MS data selection of proteins differentially regulated after 24 h of exposure to sAPPα.

Proteins differentially regulated after 24 h					
Protein	Reg.	References	Protein	Reg.	References
**APP and AD related**			**Receptors, channels, and transporters**		
SH3KBP1	Up	Rosenthal et al., 2012	SRI	Up	Genovese et al., 2020
APOE	Up	Saunders et al., 1993	GABBR1	Up	Terunuma et al., 2014
CD200	Up	Varnum et al., 2015	KCTD8	Down	Seddik et al., 2012
PICALM	Down	Zhao et al., 2015	GRIP1	Up	Tan et al., 2015
TFRC	Up	Zhou et al., 2017	GRIPAP1	Up	Chiu et al., 2017
BLMH	Down	Lefterov et al., 2000	FMR1	Up	Telias, 2019
COPS5	Up	Wang et al., 2013	FXR1	Up	Guo et al., 2015
FBXO2	Up	Atkin et al., 2014	FXR2	Up	
PCSK1	Up	Maftei et al., 2019	SLC17A6	Up	Hu et al., 2020
VPS26A	Up	Lin et al., 2015	SLC25A22	Up	
**Synaptic vesicle**			SLC41A3	Down	
SYT11	Up	Shimojo et al., 2019	SLC6A17	Down	
SNAP29	Up	Pan et al., 2005	ATP1A1	Up	
CLTA	Up	Redlingshöfer et al., 2020	ATP1A3	Up	Mata and Sepulveda, 2010;Larsen et al., 2016
RIMS1	Up	Lonart, 2002	ATP2A2	Up	
AP1G1	Up	Guardia et al., 2018	ATP2B4	Up	
RAB5A	Up	de Hoop et al., 1994	ATP6V0A1	Up	
WDR7	Up	Chen et al., 2020c	**Actin and microtubule dynamics**		
VAPA	Down	Lindhout et al., 2019	TUBB4A	Down	Sase et al., 2020
SNX12	Up	Zhang et al., 2018	TUBB4B	Down	Muñoz-Lasso et al., 2020
SNX2	Up		MAP2	Up	Johnson and Jope, 1992
**Neurite outgrowth and synapse**			MAP1S	Down	
CBLN1	Up	Yuzaki, 2011	MAPRE3	Up	Bodakuntla et al., 2019
NLGN2	Down	Katzman and Alberini, 2018	STMN3	Up	Uchida and Shumyatsky, 2015
EXOC4	Down	Liebl et al., 2005	WDR47	Up	Chen et al., 2020c
CD47	Up	Lehrman et al., 2018	CORO1A	Down	Jayachandran et al., 2014
PSD95	Down	Coley and Gao, 2018	CORO1B	Up	
AGO2	Up	Rajgor et al., 2018	CORO2A	Up	Heo et al., 2018
TWF2	Up	Yamada et al., 2007	CORO2B	Down	Chen et al., 2020b
ADD2	Up	Babic and Zinsmaier, 2011	TNIK	Down	Fu et al., 1999
NFASC	Up	Pourhoseini et al., 2021	PFDN1	Down	Liang et al., 2020
SPTBN4	Down	Machnicka et al., 2014	PFDN4	Up	
KALRN	Up	Mandela and Ma, 2012	PFDN6	Up	Gu et al., 2008
NDEL1	Down	Kuijpers et al., 2016	ACTR3B	Down	Spence et al., 2016
WASF1	Up	Derivery et al., 2009	ARPC1A	Up	
WASF3	Down		ARPC1B	Up	
WASHC5	Down	Freeman et al., 2013	ARPC5	Up	
RAC1	Up	Haditsch et al., 2009	**Proteasome and lysosome**		
RAC3	Up	de Curtis, 2019	LAMP1	Up	Hwang et al., 2019
RALA	Up	Li et al., 2007	LAMTOR1	Up	Malek et al., 2012
RALB	Up		UBQLN1	Up	Hiltunen et al., 2006
RANBP1	Up	Mignogna and D’Adamo, 2018	UBQLN2	Up	Renaud et al., 2019
RANBP3	Down		USP10	Up	Lim et al., 2019
RANBP9	Down		USP24	Up	Young et al., 2019
IGFBPL1	Up	Guo et al., 2018		–	–
PTPRS	Up	Yu et al., 2018	–	–	–

Table 5 shows the protein differentially expressed at 24 h under the categories (i) *APP and AD related*, (ii) *neurotransmitter and ion channels*, (iii) *synaptic vesicle*, (iv) *actin and microtubule dynamics*, (v) *neurite outgrowth and synapse*, and (vi) *proteasome and lysosome*.

## Discussion

This study investigated how the neuroprotective protein, sAPPα, changes the gene expression profile of a human neuron in culture. The key reagent for the study was a recombinant sAPPα (Turner et al., 2007) derived from the APP^695^ isoform, which is the predominant form in the brain. We confirmed by immunocytochemistry that APP was expressed in the neurons in culture and thereby physiologically important in this human cell culture model. The isogenic human iPSC-derived glutamatergic neurons (i^3^N) grown in culture for the studies expressed neuronal markers of axons, dendrites and pre and post synaptic compartments. The changes in the expression profile of the neurons in response to sAPPα was determined by discovery transcriptomics and proteomics.

### Transcriptome

To access the immediate effects of sAPPα on expression at the genome the time points of 30 min and 2 h after sAPPα application were chosen, resulting in 204 and 103 differentially expressed protein coding transcripts respectively. Protein coding transcripts made up 37 and 25% of the differentially expressed transcripts at 30 min and 2 h of exposure to sAPPα, respectively – the remainder were predominantly lncRNA transcripts (∼40%). With less published literature on their functions, a preliminary investigation of how they might relate to changes in neurological functions of the neurons did not give any close linkages, and so were not further investigated here. They will be the subject of a more detailed future investigation. It found that IEGs/transcription factors were a significant feature of the 30 min transcripts. A secondary expression of a cascade of genes regulated by the transcription factors will be initiated. Previously, we have shown if an IEG (e.g., *EGR-1* or *FOS*) is expressed as an mRNA by 30 min from a stimulatory induction, then upregulation of the proteins encoded by these transcripts occurs at 1–2 h (Abraham et al., 1991; Demmer et al., 1993).

The STRING functional network analysis also revealed distinct **hubs** of transcripts that encode proteins which are functionally associated to one another according to known and predicted interactions as well as from the literature. Of these

(i) ***APP*** was directly linked to five genes and each of those genes were also linked to other genes (number in parenthesis), *CX3CL1* (2), *TTR* (3), *C3* (3), *NRGN* (1), and *FOS* (15).
(ii) ***FOS*** was the largest hub and 15 other protein encoding genes were linked to it; *SERPINE1, EGR1, EGR2, PPP1R1B, HNF1A, CCNA2, KLF10, AVPR1A, CXCL1, GHRH, NOS1, NR4A3, NPAS4, RORC*, and *NFATC2* plus *APP*.
(iii) ***EGR1*** had 13 protein encoding genes linked to it, *SERPINE1, OAS3, CXCL1, BHLHE40, GDF15, NR4A3, NPAS4, EGR2, IFITM2, RRAD, NFATC2, NAB2*, and *FOS*.
(iv) ***EGR2*** was linked to eight protein encoding genes, *EGR1, WNT1, NFATC2, NPAS4, NR4A3, RRAD, FOS*, and *NAB2*.
(v) ***WNT1***, *10A, 2* and *4* collectively were linked to 21 other protein encoding genes, *EGR2, WNT10A, WNT4, MFRP, NKX2-2, RSPO3, TCF7, ATOH1, RSPO1, LRP5L, SFRP4, FZD4, SOST, MFRP, EDAR, WNT1, WNT2B, FOXL1*, and *CYP11A1*.

The hub proteins encompass the IEG transcription factors (*FOS, EGF1*, and *EGF2*) and key signalling molecules *APP* and *WNT1*, and the connected proteins fit broadly into the following functional groups of cell signalling, actin and tubulin dynamics – cytoskeleton, early response genes and transcriptional regulators, extracellular matrix and cell adhesion, and receptors, and channels and neurotransmitters.

### Proteome

The differential protein profiles measured in this study indicate that some are associated with the observed changes in gene expression of the transcription factors, that would modify the phenotype of the neurons. These long-term physiological actions of sAPPα (LTP facilitation, neurite outgrowth, neuroprotection, and gene expression) can have rapid onset (min to h) but can also be long lasting (days to months). After 2 h exposure to sAPPα in this study there were only 21 proteins differentially expressed at a stringency of *p* < 0.01 and with a fold change of 1.5 or greater compared with untreated cells (0 h). By contrast 320 and 380 differentially regulated proteins were identified at the same stringency and at 6 and 24 h, respectively. IEGs known to be involved in memory formation, which were differentially expressed after 30 min included Early Growth Factor 1 (EGR1, aka Zif268, and Krox24). SNAP 29 and PSD-95 are downstream genes modulated by EGR1 (Duclot and Kabbaj, 2017), and these were identified in our proteome profile as differentially regulated after 24 h. WNT signalling related molecules are also enriched in these data. It is interesting that APP has recently been identified as a WNT signalling receptor (Liu et al., 2021).

### Proteins Related to Neurological Function at the Synapse

#### Synaptogenesis

The long-term aim of the focus on sAPPα is that it has potential for development as a therapeutic for AD. Proteins from four risk factor genes for AD are differentially regulated in the proteome dataset (APOE, PICALM, BINI, and SH3KBP1) (Rosenthal et al., 2012). For example, BIN 1 (upregulated) is a late onset AD risk factor gene that aids memory consolidation and regulates presynaptic neurotransmitter release (De Rossi et al., 2020). Synaptogenesis is an important component of this. Synaptogenesis involves actin nucleation and encompasses WASH/WAVE/Arp2/3 proteins/Coronins/ACTR3B/AP3B1/ARPC1A, which are all differentially regulated in our study. Arp2/3 complex is necessary for the recruitment of AMPA receptors in newly formed excitatory glutamatergic synapses (Spence et al., 2016). Coronins are actin binding proteins that can regulate actin dynamics by coordinating the activities of the actin-polymerising nucleator Arp2/3 complex (Chan et al., 2011). Interestingly, CORO2A is one of the long-lived proteins (LLP) identified as having an extraordinarily long (months/years) half-life at the synapse and they are postulated to be important for long term stable memories (Heo et al., 2018). Cerebellin 1 (CBLN1, upregulated) is a synaptic organiser involved with the precise apposition of presynaptic and postsynaptic regions, essential for the formation of functional synapses. CBLN receptors are the presynaptic, neurexin (NRX) and the post synaptic, glutamate receptor (GluD2) (Yuzaki, 2011). PSD-95 (downregulated) is a postsynaptic scaffolding protein that plays a critical role in synaptogenesis and synaptic plasticity by providing a platform for the postsynaptic clustering of crucial synaptic proteins (Coley and Gao, 2018).

#### Exocytotic Release of Neurotransmitters at the Synapse

Exocytosis is a critical component of the function of the synapse. NIPSNAP1 is part of the SNARE complex (downregulated at 6 h) involved in the exocytotic release of neurotransmitters during synaptic transmission (Antonucci et al., 2016). At 24 h, SNAP29, which is associated with the disassembly of the SNARE complex (Pan et al., 2005) is upregulated. At the synaptic active zone vesicle exocytosis is also regulated by RIMS1 (RAB3, upregulated) (Lonart, 2002) and VAPA (upregulated) (Lindhout et al., 2019) while EPN1 (downregulated) is involved with endocytosis (Jakobsson et al., 2008). Syntaxin 7 (STX7) is involved with the replenishment of readily releasable synaptic vesicles in the hippocampus, which allows for sustained neurotransmitter release (Mori et al., 2021). The exocyst complex is a multiple protein complex essential for targeting exocytic vesicles to specific docking sites on the plasma membrane. For example, in *Drosophila* SEC8 (EXOC4) is involved in the regulation of synaptic microtubule formation, regulation of synaptic growth and glutamate receptor trafficking (Liebl et al., 2005).

#### Wider Transport of Cargos Important in Neurological Function

Sorting Nexins (SNX) are important for correct intracellular transport including APP and AP-cleaving enzymes, dysregulation of which causes neurodegenerative disease (Zhang et al., 2018).

NudE Neurodevelopment Protein 1 Like 1 (NDEL, downregulated) is concentrated at the axon initial segment where it regulates dynein and the trafficking of transport cargos specifically bound for the axon (Kuijpers et al., 2016). GRIP1 (glutamate receptor-interacting protein), upregulated at 6 and 24 h, regulates the homeostatic shuttling of AMPARs between cytoplasmic and synaptic pools (Tan et al., 2015). A related protein GRIPAP1 (GRIP1-associated protein 1, also known as GRASP1) is upregulated at 24 h and is involved with endosomal recycling of AMPA receptors (Chiu et al., 2017). Members of the solute carrier family (SCL) are differentially expressed both in the transcriptome and proteome and are responsible for the transport of molecules across the cell membrane (Hu et al., 2020). Members of the Na^+^ /K^+^ ATPase family (ATP1 and ATP2) controlling extracellular K^+^ and suggested to work in concert with the glutamate transporter are also differentially expressed both in the transcriptome and proteome data (Larsen et al., 2016).

### Comparison With Other Molecular Studies Investigating the Effects of Secreted Amyloid Precursor Protein Alpha

Secreted amyloid precursor protein alpha promotes the expression of the IEG encoding the plasticity protein, ARC, in primary hippocampal neurons and that in turn regulates AMPA receptor synthesis and trafficking (Livingstone et al., 2019, 2021). Many attempts to identify the sAPPα receptor have finally provided evidence of two receptors that likely are the first points of contact for sAPPα functions. Its contact through an N terminal domain with GABA_β_R1a regulates the function of the receptor to modulate synaptic transmission and plasticity (Rice et al., 2019). The C terminal 16 amino acids that differentiate sAPPα from sAPPβ act to potentiate the nicotine acetyl choline receptor, a7-nAChR, that functions in facilitating LTP (Richter et al., 2018).

Two key studies with sAPPα and global gene expression have been carried out to date. Stein et al. (2004) showed that sAPPα increased the expression of several neuroprotective genes and protected organotypic hippocampal cultures from amyloid beta–induced tau phosphorylation and neuronal death. Transthyretin was highly expressed and necessary for sAPPα protection. (Ryan et al., 2013) measured time dependent changes in the expression of transcripts in organotypic rat hippocampus slice cultures in response to sAPPα as measured by an Affymetrix rat gene array. They showed chronological changes from 15 min to 2 h to 24 h with many unique changes at each time point and small numbers of overlaps. Most of the expression was upregulation at 15 min but downregulation predominated at the other two time points. Consistent with our study, IEGs/transcription factors were prominent in the upregulated genes (for example, FOS and EGR-1), as well as NFk-B and CREB regulated genes, whereas at 2 h, inflammatory response regulation was the most significant biological function of the highest scoring network. A recent mouse proteomic study comparing an AD model and healthy controls identified protein members for similar family group as this study (splicing factors, ribosomal proteins, proteasome, chaperones, syntaxins, and solute carrier proteins) (Elder et al., 2021). A genome wide association study (GWAS) identifying AD risk genes highlighted 11 causal genes, 4 of which were found in this study [sortin, nexin, syntaxin, and pleckstin homology domain proteins (Wingo et al., 2021)]. Our findings and those from Ryan et al. (2013) are consistent with a broader study of amyloid precursor protein family members in the adult cortex (Aydin et al., 2011).

### Limitations of the Study

The transcriptome and proteome outputs are samplings of very large datasets, and the setting of relatively high stringency will inevitably lead to some relevant data being removed from the analysis as false negatives. Proteome analysis relies on the analysis of the intensity of peaks that are affirmed in technical replicates within statistical boundaries. This means proteins present at low levels tend to show lower reproducibility and often fail statistical significance. In this case ∼3,200 proteins passed our stringent filters were analysed for differential regulation between the time points. Nevertheless, the advantages of the analytical tools facilitate a grouping of the individual protein data through known functional interactions, and functional annotation through the three main GO aspects of Biological Process, Molecular Function and Cellular Component giving an integrated picture of what features of the neuron are being affected. While validation of the many individual changes seen in both transcripts and proteins throughout their time courses would be desirable by independent methods such as quantitative PCR for the transcripts, and immunocytochemistry and Western analysis for the proteins, these methods are applicable in reality to a very small proportion of the differentially expressed molecules of the data set. Indeed Aebersold et al. (2013) have argued that targeted proteomic methods provide a better method of validation than Western blotting. Here, the study aimed to establish a chronological global profile of a gene expression changes and to determine from the collective data whether dynamic physiological changes could be derived.

### Future Directions

This study has established small and large molecular hubs that are activated in responses to sAPPα and these will be rich sources for elucidating the detailed molecular pathways of the sAPPα mediated effects on phenotype of the neuronal cells like memory enhancement (Meziane et al., 1998), neuroprotection (Turner et al., 2007), neurogenesis (Caille et al., 2004; Baratchi et al., 2012), and neurotrophy (Chasseigneaux et al., 2011). Recombinant sAPPα also enhances LTP (Mockett et al., 2019) and spatial learning (Ishida et al., 1997; Taylor et al., 2008). sAPPα is protective against memory impairments (Fol et al., 2016; Tan et al., 2018) but the details of how that occurs through the myriad of signalling pathways is still to be elucidated. Understanding these detailed mechanisms of how sAPPα mediates it global functions is important if the molecule is to be exploited for its therapeutic potential.

This study of the way in which sAPPα influences the gene expression profile specifically of the human neuron to fulfil its protective and growth roles has shown there is a comprehensive response in the proteins that are related to the functions of neurological significance. From the initial direct interaction of sAPPα with cellular components there is a large ripple effect that spreads to affect broadly the wider function of the neuron and to change its character.

## Data Availability Statement

The original contributions presented in the study are publicly available. This data can be found here: GEO submission GSE197725 and the ProteomeXchange Consortium *via* the PRIDE repository PXD031704 (Perez-Riverol et al., 2022).

## Author Contributions

KP helped plan the research, carried out the experimental work, and co-wrote the manuscript. WT supervised the project and co-wrote the manuscript. SH co-supervised the project and assisted with the writing of the manuscript. TK managed the proteome aspect of the study and its analysis and assisted with the writing of the manuscript. OJ provided the electrophysiology experimental expertise to validate the human neurons used for the study. All authors contributed to the article and approved the submitted version.

## Conflict of Interest

The authors declare that the research was conducted in the absence of any commercial or financial relationships that could be construed as a potential conflict of interest.

## Publisher’s Note

All claims expressed in this article are solely those of the authors and do not necessarily represent those of their affiliated organizations, or those of the publisher, the editors and the reviewers. Any product that may be evaluated in this article, or claim that may be made by its manufacturer, is not guaranteed or endorsed by the publisher.

## Footnotes

1
http://string-db.org

2
https://www.ncbi.nlm.nih.gov/

## Supplementary Material

Supplementary Figure 1 Differentially regulated proteins at 6 h. STRING functional network analysis illustrating that >80% of the differentially expressed proteins [log2 (FC) ± 0.58, *p* ≤ 0.01] in the 6 h SWATH-MS data set fit into one or more enriched Gene Ontology (GO) group. Diagram shows proteins and their relationship to others when searched against the human protein database. Proteins (nodes) are coloured to represent enriched GO terms. Biological processes GO terms: red, cytoskeletal organisation; purple, membrane organisation; light green, intracellular transport; yellow, protein localisation; pink, immune response activating cell signalling pathways; dark green, gene expression. Cellular Component GO terms: teal, vesicle; orange, mitochondria; mauve, synapse; brown, axon.

Supplementary Figure 2 Differentially regulated proteins at 24 h. STRING functional network analysis illustrating that >80% of the differentially expressed proteins [log2 (FC) ± 0.58, *p* ≤ 0.01] in the 24 h SWATH-MS data set fit into one or more enriched Gene Ontology (GO) groups. Diagram shows proteins and their relationship to others when searched against the human protein database. Proteins (nodes) are coloured to represent enriched GO terms. Biological processes GO terms: red, cytoskeletal organisation; purple, membrane organisation; light green, intracellular transport; yellow, protein localisation; pink, immune response activating cell signalling pathways; dark green, gene expression. Cellular Component GO terms: teal, vesicle; orange, mitochondria; mauve, synapse; brown, axon.

Supplementary Table 1 RNA sequencing data of differentially expressed transcripts at 30 min and 2 h.

Supplementary Table 2 SWATH-MS data of differentially regulated proteins at 2 h, 6 h, and 24 h.

Supplementary Table 3 SWATH-MS 6 h STRING enrichment: molecular function and biological process GO Terms.

Supplementary Table 4 SWATH-MS 24 h STRING enrichment: cellular component, molecular function and biological process GO Terms.

Supplementary Table 5 SWATH-MS identification of proteins with neuronal functions.

Supplementary Table 6 Neuronal function proteins differentially regulated at 6 h and 24 h.
