# Supplementary figures and images for: Secreted Amyloid Precursor Protein Alpha, a Neuroprotective Protein in the Brain Has Widespread Effects on the Transcriptome and Proteome of Human Inducible Pluripotent Stem Cell-Derived Glutamatergic Neurons Related to Memory Mechanisms
Source: Front Neurosci. 2022 May 26;16:858524. doi: 10.3389/fnins.2022.858524 (PMC9179159; doi:10.3389/fnins.2022.858524)

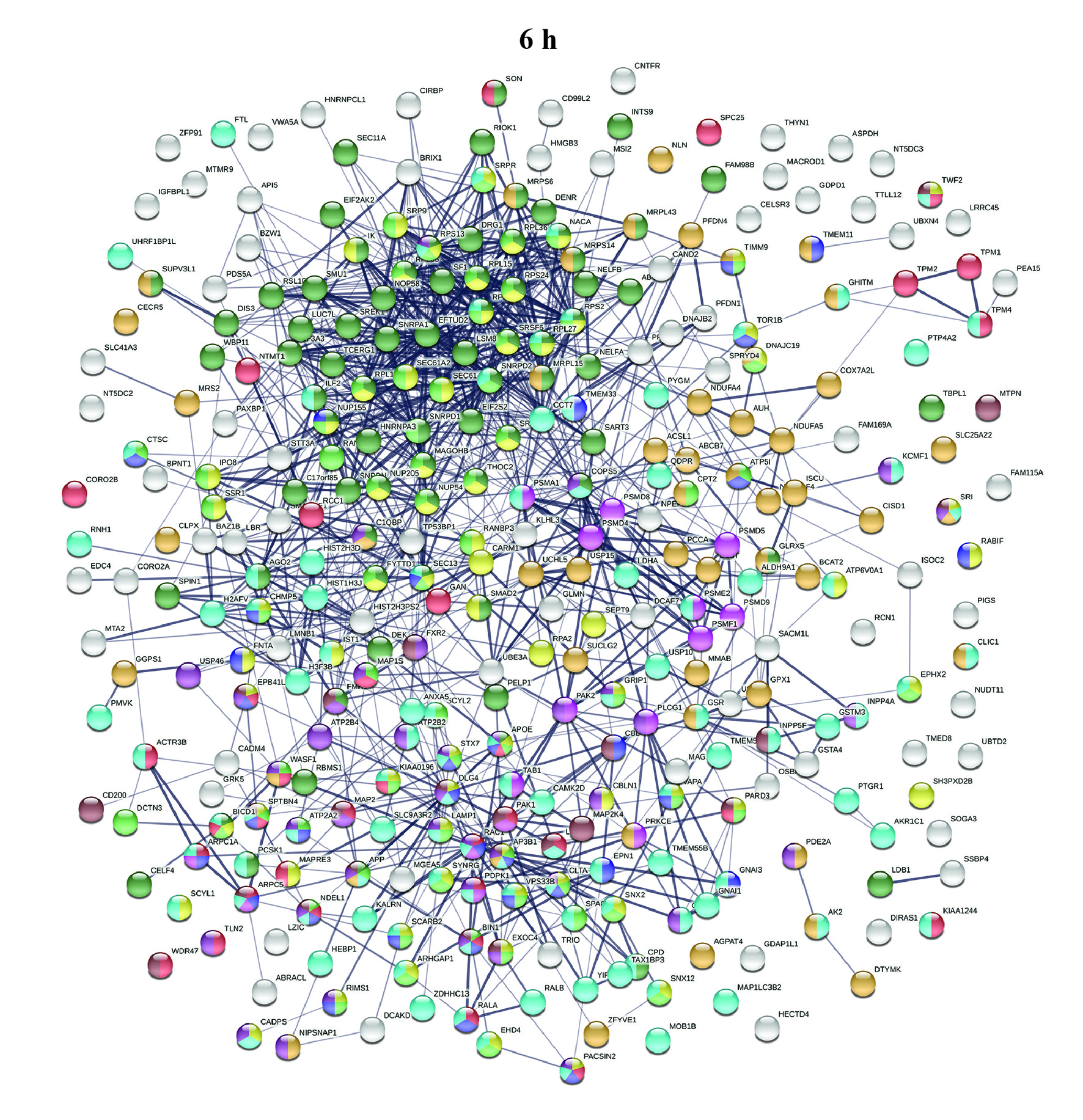

Supplement: Supplementary Figure 1 — Differentially regulated proteins at 6 h. STRING functional network analysis illustrating that >80% of the differentially expressed proteins [log2 (FC) ± 0.58, p ≤ 0.01] in the 6 h SWATH-MS data set fit into one or more enriched Gene Ontology (GO) group. Diagram shows proteins and their relationship to others when searched against the human protein database. Proteins (nodes) are coloured to represent enriched GO terms. Biological processes GO terms: red, cytoskeletal organisation; purple, membrane organisation; light green, intracellular transport; yellow, protein localisation; pink, immune response activating cell signalling pathways; dark green, gene expression. Cellular Component GO terms: teal, vesicle; orange, mitochondria; mauve, synapse; brown, axon. [file Image_1.JPEG]

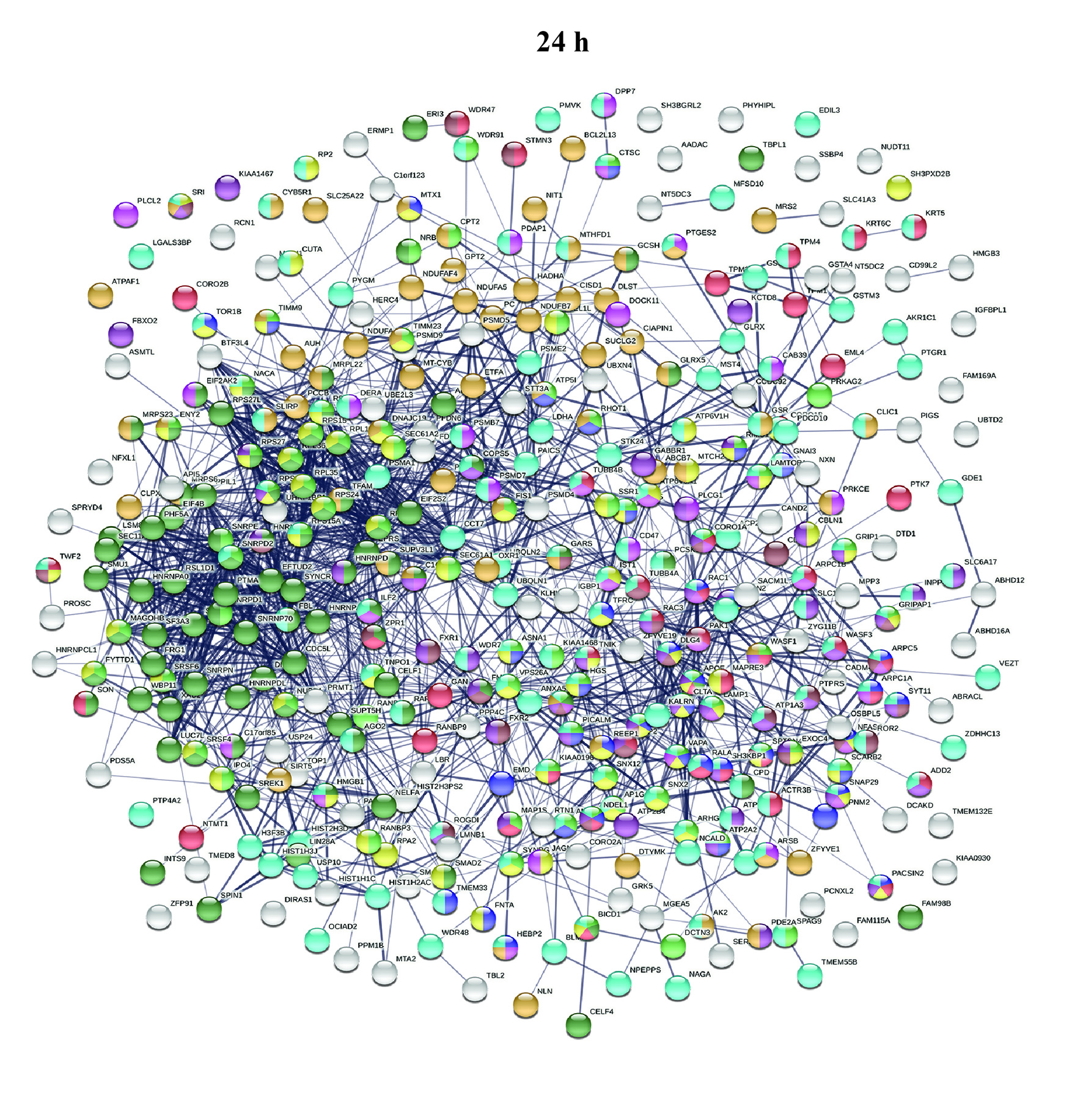

Supplement: Supplementary Figure 2 — Differentially regulated proteins at 24 h. STRING functional network analysis illustrating that >80% of the differentially expressed proteins [log2 (FC) ± 0.58, p ≤ 0.01] in the 24 h SWATH-MS data set fit into one or more enriched Gene Ontology (GO) groups. Diagram shows proteins and their relationship to others when searched against the human protein database. Proteins (nodes) are coloured to represent enriched GO terms. Biological processes GO terms: red, cytoskeletal organisation; purple, membrane organisation; light green, intracellular transport; yellow, protein localisation; pink, immune response activating cell signalling pathways; dark green, gene expression. Cellular Component GO terms: teal, vesicle; orange, mitochondria; mauve, synapse; brown, axon. [file Image_2.JPEG]
